# Effective Optical Properties of Plasmonic Nanocomposites

**DOI:** 10.3390/ma7020727

**Published:** 2014-01-27

**Authors:** Christoph Etrich, Stephan Fahr, Mehdi Keshavarz Hedayati, Franz Faupel, Mady Elbahri, Carsten Rockstuhl

**Affiliations:** 1 Institute of Condensed Matter Theory and Optics, Abbe Center of Photonics, Friedrich-Schiller-Universit¨at Jena, Max-Wien-Platz 1, 07743 Jena, Germany; E-Mails: christoph.etrich@uni-jena.de (C.E.); sfprod@gmx.de(S.F.); 2 Nanochemistry and Nanoengineering, Institute for Materials Science, Faculty of Engineering, Christian-Albrechts-Universität zu Kiel, Kaiserstrasse 2, 24143 Kiel, Germany; E-Mails: mke@tf.uni-kiel.de (M.K.H.); me@tf.uni-kiel.de (M.E.); 3 Chair for Multicomponent Materials, Institute for Materials Science, Faculty of Engineering, Christian-Albrechts-Universität zu Kiel, Kaiserstrasse 2, Kiel 24143, Germany; E-Mail: ff@tf.uni-kiel.de; 4 Institute of Polymer Research, Helmholtz-Zentrum Geesthacht, Max-Planck-Str. 1, 21502 Geesthacht, Germany; 5 Institute of Theoretical Solid State Physics, Karlsruhe Institute of Technology, Wolfgang-Gaede-Strasse 1, 76131 Karlsruhe, Germany

**Keywords:** nanocomposite, plasmonics, metamaterials, homogenization

## Abstract

Plasmonic nanocomposites find many applications, such as nanometric coatings in emerging fields, such as optotronics, photovoltaics or integrated optics. To make use of their ability to affect light propagation in an unprecedented manner, plasmonic nanocomposites should consist of densely packed metallic nanoparticles. This causes a major challenge for their theoretical description, since the reliable assignment of effective optical properties with established effective medium theories is no longer possible. Established theories, e.g., the Maxwell-Garnett formalism, are only applicable for strongly diluted nanocomposites. This effective description, however, is a prerequisite to consider plasmonic nanocomposites in the design of optical devices. Here, we mitigate this problem and use full wave optical simulations to assign effective properties to plasmonic nanocomposites with filling fractions close to the percolation threshold. We show that these effective properties can be used to properly predict the optical action of functional devices that contain nanocomposites in their design. With this contribution we pave the way to consider plasmonic nanocomposites comparably to ordinary materials in the design of optical elements.

## 1. Introduction

Plasmonic nanocomposites, as considered here, consist of metallic nanoparticles embedded in a dielectric host material [1]. Using physical vapor deposition, the nanoparticle filling factor can be varied over the whole range from the regime of isolated non-interacting particles up to above the percolation threshold where metallic behavior is observed [2]. The most prominent feature of metallic nanoparticles is their ability to sustain localized surface plasmon polaritons at discrete frequencies that are in the visible spectrum for noble metals such as gold or silver [3]. To excite plasmon polaritons an external electromagnetic field is coupled resonantly to the charge density oscillation of the free electrons in the metal, which tremendously increases the polarization of the metallic nanoparticles and, thus, the optical response [4]. Besides a significantly enhanced scattering and absorption cross-section, the local electro-magnetic field close to the nanoparticle is enhanced. These basic properties of an isolated metallic nanoparticle find many applications in sensing, imaging, catalysis or for photon management [5–8].

If metallic nanoparticles are brought together with a sufficient density in a dielectric host, they constitute a referential example of what is nowadays known as a metamaterial [9]. Metamaterials are artificial materials with tailored effective properties, such as dispersive permeability and permittivity [10]. Other more primary optical properties, such as, e.g., the polarization state or the directional transmissivity of light, can be affected, as well [11,12]. The ability to affect the permeability or dispersive permittivity requires constituting entities, so-called meta-atoms, with a strong magnetic or electric dipolar response, respectively [13]. The simplest meta-atom providing an electric dipolar response is an isolated metallic nanoparticle. In lowest order approximation, the effective permittivity of the corresponding plasmonic nanocomposite will then be represented by a Lorentzian shaped material dispersion relation around the frequency where the localized surface plasmon polariton is sustained by the individual metallic nanoparticle. This constitutes a viable route towards materials having a dispersive permittivity on demand, since the properties of all materials consistent with causality can be written as a superposition of Lorentzian resonances. Each of these Lorentzian resonances is characterized by an oscillator strength, resonance frequency and a lifetime.

Having such tailored materials at hand would significantly enlarge the degrees of freedom in designing optical devices. To be specific, nowadays, considering only naturally available materials, the optical properties of a specific optical element, such as a coating or a lens, have to be looked up in a database. Additionally, in this database, only a few discrete materials are available. This constitutes a severe limitation in the design of many optical devices. Plasmonic nanocomposites promise to overcome this limitation by their ability to provide materials with adjustable and tunable properties. However, to consider plasmonic nanocomposites in the design process of functional optical devices, it is of paramount importance to assign effective properties to these materials that can reliably predict the optical response.

The assignment of effective properties to plasmonic nanocomposites is a long standing problem and many solutions and associated theories were suggested [14–16]. The most prominent and widely used, is the Maxwell-Garnett theory [17]. Here, effective properties are assigned to a medium made from spherical inclusions in a host material, *i.e.*, in full agreement with the nominal geometry as considered here. Finally, the free parameters in the theory are the filling fraction and the intrinsic material properties of the constituents. However, although such a theory is useful for grasping the basic properties of plasmonic nanocomposites, it ceases to be reliable if the filling fraction tends to be large. In the course of time, many extensions and alternative theories were developed and applied to solve this problem, but they all have their limitations [18–22]. For example, they may either require a periodic arrangement of the metallic nanoparticles [23] or can only predict bounds for the effective permittivity [24]. Furthermore, in general, they tend to fail for excessive filling fractions, *i.e.*, filling fractions approaching the percolation threshold. However, especially plasmonic nanocomposites with a high filling fraction are instrumental for many applications [25–27]. A high filling fraction suggests a much stronger dispersion and entails that the materials be characterized by either extremely large or small permittivities. This is of paramount importance to shrink the size of many devices, e.g., considering nanocoatings. Restricting the considerations to small filling fractions, the effective permittivity of the nanocomposite largely adheres to that of the host material, and only a weak dispersion is observed. This disqualifies the material for many applications.

Here, we solve this problem by assigning effective properties to dense plasmonic nanocomposites made from amorphously arranged metallic nanoparticles using full wave simulations in the first instance [28]. This approach was pioneered in the context of metamaterials [29]. There, however, it is usually applied to strictly periodic structures, although amorphous materials were considered occasionally, as well [30]. In our approach, the rigorously calculated complex reflection and transmission coefficients from a slab of a nanocomposite are inverted to assign an effective permittivity. An effective permeability is equally obtained, but it does not deviate from unity. Thus, it is not discussed here. The rigorously retrieved effective properties are compared to predictions from effective medium theories. Moreover, we discuss the impact of different materials and show that, surprisingly, to some extent, the effective properties of plasmonic nanocomposites made from gold possess a much stronger dispersion when compared to those made of silver. We provide a coherent explanation for this observation and argue that the larger intrinsic absorption of gold eventually is beneficial to suppress the interaction of the metallic nanocomposites at high filling fractions. This interaction usually has a detrimental effect.

**Figure 1.**
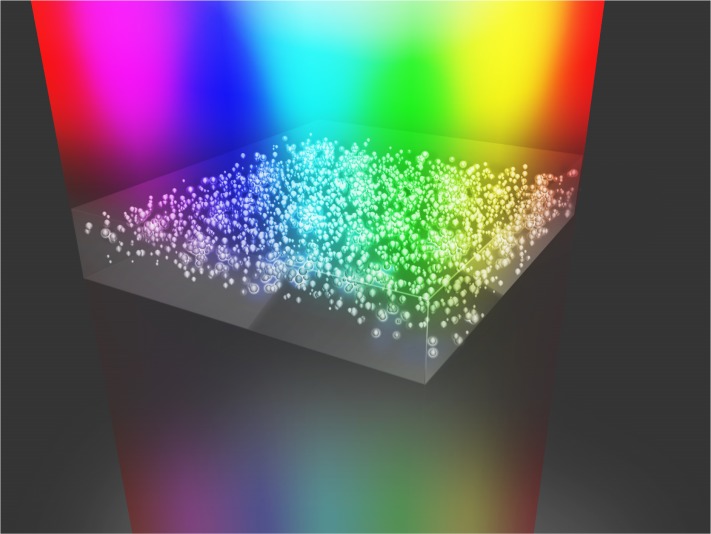
Schematic of the configuration under consideration. A plasmonic nanocomposite made from densely, but randomly, arranged metallic nanoparticles shall be an integrated part of an optical device, e.g., as an anti-reflection coating. The consideration of the entire material in an optical analysis of such a device is too complicated. Therefore, it should be described by effective optical properties to predict the reflected and transmitted light properly; as indicated in the figure.

The unique feature of our analysis is the application of large-scale computational resources. This permits us to consider extended spatial domains of nanocomposites such that their intrinsic properties are entirely reflected. The basic geometry of the considered structure is shown in Figure 1 for the purpose of visualizing the problem. For many traditional metamaterials effective material parameters cannot be assigned, due to a significant spatial dispersion [31], which is also discussed in terms of a non-locality. In contrast, here we show that the effective properties as retrieved for plasmonic nanocomposites, are useful for predicting the optical response of functional devices, considering the composites in their design. Therefore, with our work we significantly contribute to the further evolution of nanocomposites by identifying a reliable path to consider them comparably to ordinary materials in the design of optical elements.

## 2. Experimental Section

### 2.1. Description of the Plasmonic Nanocomposite

Motivated by various experimental results, we consider plasmonic nanocomposites made from silver or gold nanoparticles here [25,26,32]. They are fabricated in a SiO_2_ matrix by a suitable co-sputtering technique. The mean diameter of the nanoparticles and their filling fraction can be extracted from high resolution transmission electron microscope (HRTEM) and energy dispersive X-ray spectroscopy measurements, respectively [33]. Eventually, the deposition rate in the fabrication process is a valuable means to modify it. We consider here spherical particles with a mean diameter of 4.1 nm. Their filling fraction can be tuned. It is between 15% and 40%. The metal properties, as taken into account in the simulations, are as tabulated for bulk materials with an additional damping term, which accounts for the finite size of the nanoparticles [34,35]. This is necessary, since the spatial extent of the nanoparticles is already much smaller than the mean free path of the free electrons in the metal. This mean free path is in the order of tens of nanometers. Thus, the finite size causes an additional scattering, which reduces the lifetime of the excited localized surface plasmon polariton. SiO_2_ was considered as a nondispersive material with a permittivity of 2.25 and the material properties of Si as considered in some parts of our work, are taken from the literature [36]. To obtain an actual representation of the cluster in the numerical work, random arrangements of nanoparticles were generated. In agreement with experimental observations from TEM micrographs and motivated by the growth process of the samples, we excluded the possibility of penetrating particles.

It should be noted that the nanocomposite as considered here is simplified in two ways compared to the real structure. First, it is assumed that the nanoparticles are monodisperse, *i.e.*, their size is the same. This assumption, however, is not severe since in the quasi-static approximation (applicable if the particle size is much smaller than the wavelength) the resonance frequency is independent of the size of the nanoparticle. Only the magnitude of the polarizability changes. However, this effect cancels in the effective properties exactly with an increasing absolute number of nanoparticles necessary to achieve a given filling fraction. Therefore, it is not surprising that in selected simulations accounting for the exact size-dispersion of the nanoparticles, extracted from measurements, we obtained an identical response compared to simulations with a constant particle size. However, we would like to stress that the assumption or the respective violation of the monodisperse character affects the observation of the actual percolation threshold in experiments. The percolation threshold, *i.e.*, the onset of a significant conduction, occurs for monodisperse metallic nanoparticles for a filling fraction of about 15%, whereas it is much higher for polydisperse metallic nanoparticles [2]. For the present samples it is approximately 15%. Here, we wish to stress that in our simulations we always stay below the percolation threshold since the nanoparticles are assumed to be nontouching, *i.e.*, each nanoparticle is isolated.

Second, a strictly spherical shape is assumed. This, of course, is not entirely correct but it is nevertheless a reasonable approximation. Due to the minimization of the surface energy, the nanoparticles always grow as spheres. We only observe deviations from spherical shape at higher filling fractions if two growing particles approach each other. Moreover, the consideration of more complicated geometries for each nanoparticle is cumbersome since only marginal information is available on their precise shape. However, we wish to stress that the different shapes will introduce a dispersion in the resonance frequencies of the polarizabilities of the individual nanoparticles [37]. However, as shown below, such an effect occurs in nanocomposites also, due to the strong coupling of neighboring nanoparticles. If we assume that the change in the resonance frequency due to nearest neighbor coupling is larger than the change in the resonance frequency due to a different geometry, the results should be reliable. Nevertheless, this assumption has to be kept in mind while comparing simulations to experimental results.

### 2.2. Finite-Difference Time-Domain Method

For our simulations, the finite-difference time-domain (FDTD) method [38] was used. It discretizes Maxwell’s equations in space and time on a so-called Yee grid and employs a time advancing algorithm to simulate the evolution of an incident field through the structure of interest. The composite is considered in a periodically arranged supercell with a size of 100 nm *×* 100 nm in the lateral directions. The size in the vertical direction of the plasmonic nanocomposite is varied according to the specific computational situation. In the principal propagation direction of the incident field perfectly matched layers are applied to consider an infinitely extended space. The spatial discretization is 0.3 nm. The time was discretized within the limits of the Courant criterion. Material properties are taken into account by means of auxiliary differential equations. Even though FDTD simulations are performed in the time domain, which in general permits to the extracting of a frequency response from one simulation, we performed individual simulations at each wavelength to adjust the material properties according to the experimental values. As illumination, we used a linearly polarized plane wave at normal incidence. A linear polarization is fully sufficient, since no preference for the polarization is expected, because the sample is amorphous and made from isotropic constituents.

**Figure 2.**
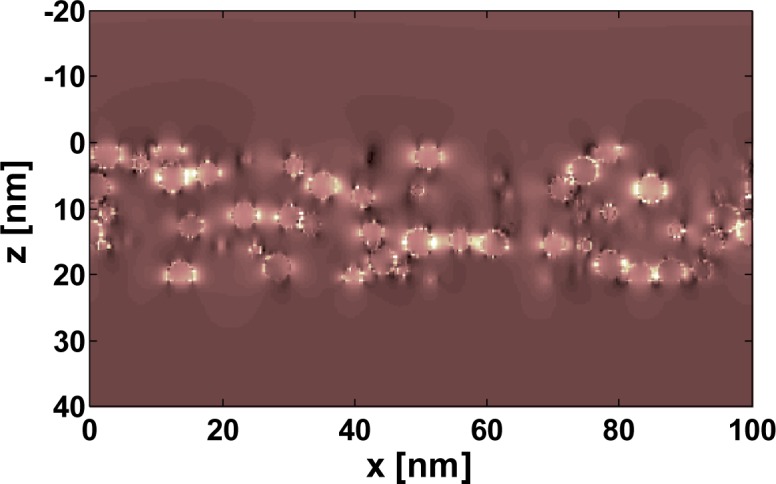
Amplitude of the electric field in a cross section through the plasmonic nanocomposite (between z = 0 nm and z = 20 nm). The incident linearly polarized plane wave propagates in the +z-direction. The plasmonic nanocomposite has a filling fraction of 20% and consists of randomly arranged non-touching silver nanospheres with a diameter of 4.1 nm embedded in a generic mondisperse dielectric material (*ϵ* = 2.25). The amplitude is shown at an incident wavelength of 430 nm.

A selected snapshot of the amplitude of the electric field in a cross-section through a referential silver plasmonic nanocomposite is displayed in Figure 2. The incident wavelength of 430 nm corresponds to the resonance wavelength of the isolated nanoparticle, as indicated by the large and constant field amplitude inside the nanoparticles. All nanoparticles are excited to a comparable extent. We also observe hot spots at selected sites whenever a multiple number of nanoparticles is closely spaced. Here capacitive coupling takes place between neighboring nanoparticles which enhances the resonance strength and also redshifts the resonance wavelength. The effect is well studied for dimers or periodic arrays of nanoparticles and occurs also in an amorphous arrangement, as considered here [39–41]. These additional resonances are important, as will be seen below, since they strongly affect the effective properties of the nanocomposite. Moreover, these coupling events are not considered in most of the ordinary homogenization theories.

To extract the complex reflection and transmission coefficients through a slab of the nanocomposite, the total electric field in a plane directly above or below was spatially averaged. Whereas this provides the amplitude of the transmission coefficient directly, the reflection coefficient was calculated by subtracting the incident field prior to spatial averaging. This incident field was obtained in a supporting simulation where the propagation of the illumination in the absence of the sample was simulated. It should be noted that the reflected and transmitted amplitudes are non-zero, within numerical precision, only for the electric field component of the linearly polarized illumination. No depolarization was observed. This strongly supports the claim of considering the plasmonic nanocomposite effectively as an isotropic material.

#### 2.2.1. Homogenization

To assign effective properties to the plasmonic nanocomposite, we invert the complex reflection *R*(*ω*) and transmission *T*(*ω*) coefficients as analytically calculated for a slab with thickness *d*, embedded in air and made from a homogenous, isotropic, dispersive material [29]. This provides analytical expressions for the effective refractive index *n*(*ω*) and the impedance *Z*(*ω*) that are

$$\begin{array}{c} n(\omega )k_{0}d=\text{arccos }\left[\frac{1}{2T(\omega )}(1-R{\left(\omega \right)}^{2}+T{\left(\omega \right)}^{2})\right]+2m\pi ,\\ Ζ(\omega )=\pm \sqrt{\frac{{\left(1+R\;(\omega )\right)}^{2}-T{\left(\omega \right)}^{2}}{{\left(1-(\omega )\right)}^{2}-T{\left(\omega \right)}^{2}}}. \end{array}$$

Here, *k*_0_ = 2*π/λ*_0_ is the wave number of the free space wavelength. These effective wave parameters, as they are usually called, are linked to the effective material parameters permittivity *ϵ*(*ω*) and permeability *μ*(*ω*) assuming again a homogenous, isotropic, dispersive material by ϵ(*ω*) = *n*(*ω*)*/Z*(*ω*) and *μ*(*ω*) = *n*(*ω*)*Z*(*ω*).

To compare the obtained results to analytical predictions from traditional homogenization theories, we use a Maxwell-Garnett effective medium theory. There, the effective permittivity is calculated according to:

$$\frac{\epsilon (\omega )-\epsilon_{1}(\omega )}{\epsilon (\omega )+2\epsilon_{1}(\omega )}=f\frac{\epsilon_{2}(\omega )-\epsilon_{1}(\omega )}{\epsilon_{2}(\omega )+2\epsilon_{1}(\omega )}$$ (1)

with *f* being the volume filling fraction of the medium described by subscript 2 in a background material indicated by subscript 1.

## 3. Results and Discussion

**Figure 3.**
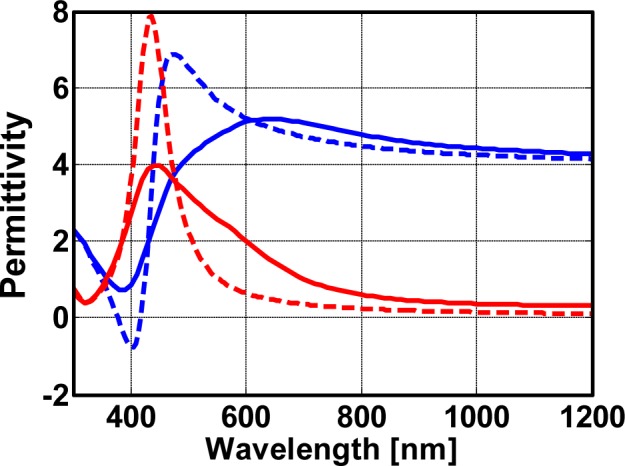
Effective properties of a plasmonic nanocomposite with a filling fraction of 20%. The effective properties are calculated by means of full wave finite-difference time-domain (FDTD) simulations (solid lines) and a Maxwell-Garnett effective medium theory (dashed line). The real part of the effective permittivity is shown in blue whereas the imaginary part is shown in red.

Referential results of the effective properties for a plasmonic nanocomposite made from silver nanoparticles are shown in Figure 3. Here, we consider a plasmonic nanocomposite with an intermediate filling fraction of 20%. We distinguish between effective properties retrieved from full wave simulations (solid line) and obtained by means of the Maxwell-Garnett effective medium theory (dashed line).

In both cases, a strong dispersion of the effective permittivity can be seen around a resonance wavelength close to 420 nm. This resonance wavelength is best extracted from the peak in the imaginary part of the effective permittivity. However, resonance strength, as well as line width strongly deviate among both methods. We would like to stress that the correct properties are those retrieved from the full wave simulations. Only these parameters correctly predict the actual reflection and transmission from a slab. The strength of the resonance of the true effective properties is approximately half the strength of the resonance predicted using the effective medium theory. Moreover, the line width of the true effective properties is equally enlarged by approximately a factor of two. An exact quantification, however, is not possible, since we clearly see in Figure 3, most notably in the imaginary part of the permittivity, that the dispersion of the permittivity as retrieved from full wave simulations shows a multi-hump structure. This suggests that the dispersion cannot be described by an individual Lorentzian, but rather, multiples thereof. This is a feature that can be easily explained by the emergence of strongly coupled clusters of nanoparticles inside the nanocomposite where an integer number of nanoparticles group together. These clusters induce resonances at different wavelengths if compared to the resonance wavelengths of isolated nanospheres. Examples for such clusters can be seen in Figure 2, where they have been discussed as being responsible for causing hot spots.

Simultaneously with the much broader resonances, also, a spectral region of anomalous dispersion was encountered, where the real part of the permittivity increases with the wavelength. This is in contrast to an ordinary dispersion which suggests a decreasing permittivity with an increasing wavelength. We would like to stress that such an anomalous dispersion is a generic feature of all resonances and not unique to the material under consideration, although the spectral domain is extraordinarily large. Again, it is large, because the dispersion is caused not just by a single Lorentzian oscillator, but rather multiples thereof with slightly detuned resonance wavelengths.

From the quantitative difference of the effective properties as retrieved with the different methods we can conclude that the Maxwell-Garnett theory is not applicable anymore for these dense nanocomposites. Futuremore, an *ad hoc* modification of the free parameters will not allow for obtaining an agreement. To retrieve the effective properties of these dense nanocomposites full wave simulations are necessary. Only by means of such simulations, as shown below, it will be possible to properly predict the optical response of functional devices. It should be noted that the discussion here for nanocomposites with a filling fraction of 20% applies to all other filling fractions studied. Quantitative differences, of course, are less severe for lower fractions but remain notable.

**Figure 4.**
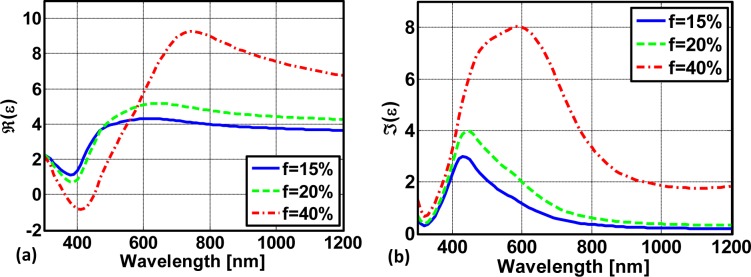
Rigorously calculated effective properties of a silver nanocomposite depending on the filling fraction. (**a**) The real part and (**b**) imaginary part.

The effective properties of silver nanocomposites depending on the filling fraction are shown in Figure 4. We distinguish between the real part in Figure 4a and the imaginary part in Figure 4b. The filling fractions shown correspond to 15%, 20% and 40%. We clearly see that with an increasing filling fraction the resonance wavelength shifts to longer wavelengths. This is in agreement with observations from periodically arranged metallic nanoparticles [40] but it is also observed in amorphous samples [42]. The coupling is dominated by the interaction of horizontally coupled metallic nanoparticles. This can be easily explained by considering the fact that horizontally coupled particles strongly perturb the fields of the eigenmode associated with the dipolar resonance of the metallic nanoparticle mutually. The field of this eigenmode concentrates in an equatorial plane of the nanoparticle. Hence, perturbing these spatial domains where the modes tend to localize with a neighboring nanoparticle affects the eigenenergies of the mode strongest. The perturbation is less severe if the particles are vertically coupled, *i.e.*, if they are aligned in chains along the propagation direction.

Considering excessively large filling fractions, *i.e.*, 40% in our case, we clearly observe a significant deviation from the Lorentzian resonance shape. Particularly, a plateau is formed in the imaginary part where a constant value is obtained in an extended spectral domain. Moreover, the regime of anomalous dispersion is largely enhanced. Values for the permittivity as large as nine are obtained. The induced dispersion is so strong that the nanocomposite will effectively have metallic properties in a finite spectral domain.

**Figure 5.**
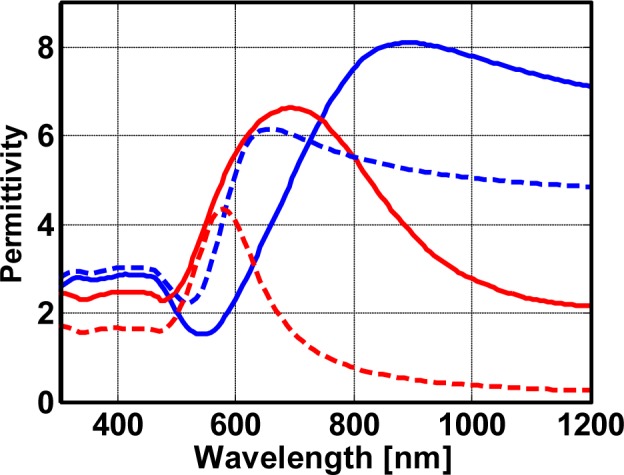
Effective properties of a gold nanocomposite with a filling fraction of 20%. The effective properties are calculated by means of full wave FDTD simulations (solid lines) and a Maxwell-Garnett effective medium theory (dashed line). The real part of the effective permittivity is shown in blue whereas the imaginary part is shown in red. The plasmonic nanocomposite consists of randomly arranged non-touching gold nanospheres with a diameter of 4.1 nm embedded in a generic nondispersive dielectric material (*ϵ* = 2.25).

The analysis discussed above for nanocomposites made from silver can be carried out with nanocomposites made from gold as well. Selected results in terms of effective properties for a nanocomposite with a 20% filling fraction are presented in Figure 5. The results are similar to the case of nanocomposites made from silver, with three notable differences. The first is of obvious nature and concerns the shift of the resonance towards longer wavelengths at around 650 nm. This is reminiscent of the fact that the plasma frequency of gold is lower and the dipolar resonance of small gold nanoparticles is shifted towards longer wavelengths. The second is the complicated functional dependency of the effective properties of the composite at a wavelength lower than approximately 510 nm. However, this is only a trace from the intrinsic material properties of gold, which also no longer follows a Drude type dependency at such low wavelengths.

The most interesting feature of the analysis are the larger values in the achievable effective permittivity if compared to nanocomposites made from silver with the same filling fraction. The imaginary part, where those aspects are usually best perceived, is larger by 50%. This is surprising, since common wisdom usually suggests that silver is the preferable plasmonic material, because its intrinsic absorption is much less than that of gold. This implies a much better fulfillment of the Fröhlich condition, a larger value of the polarizability in resonance for otherwise identical systems and, hence, a much stronger dispersion. However, it also seems that the coupling to neighboring nanoparticles is enhanced. This is detrimental for the effective properties. The spread in resonance wavelengths of eigenmodes supported by the nanocomposite eventually limits the achievable maximal value for the permittivity. In contrast, if the particles are less vulnerable against such an interaction, since non-radiative losses dominate over radiative losses, the maximal achievable values for the dispersion of the nanocomposite is larger. This holds for the situation under consideration, since gold has a much larger imaginary part in its intrinsic permittivity than silver. In summary, neighboring nanoparticles do not experience each other in the gold nanocomposite, since they tend to absorb the incident light and not to scatter it. This is an important distinction that has to be considered in choosing a suitable material for a specific application. This peculiar feature would not be observable in an effective medium description based on Maxwell-Garnett theory, as can be also seen in the figure.

After all, the retrieval of effective properties is important. However, these effective properties are only useful if they can predict the optical properties of functional devices. Motivated by recent experiments, we consider here the use of a plasmonic nanocomposite made from gold as an integrated part of a bilayer anti-reflection structure on top of a silicon substrate. The effective properties of the considered nanocomposite are shown in Figure 5. The exact layer configuration is such that a dielectric spacer (*ϵ*(*ω*) = *ϵ* = 1.69) of varying thickness is deposited on a silicon substrate. On top of the dielectric spacer, the plasmonic nanocomposite is deposited. The purpose of the structure is to suppress reflection at multiple wavelengths in the visible spectrum, such that reflection is reduced across an extended spectral range.

**Figure 6.**
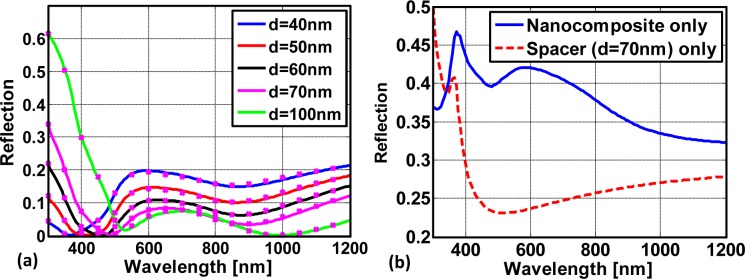
(**a**) Calculated reflection from a silicon substrate that is covered with a dielectric spacer (*ϵ* = 1.69) of various thicknesses and a layer made from a 20 nm thick gold nanocomposite. The solid lines are calculated by means of a transfer matrix algorithm, where the composite has been considered in terms of effective properties. The associated stars are the results from rigorous FDTD simulations taking the plasmonic nanocomposite fully into account; (**b**) for referential purpose we also show the reflectance from the silicon substrate with the nanocomposite alone and with only a dielectric spacer of thickness of 70 nm.

**Figure 7.**
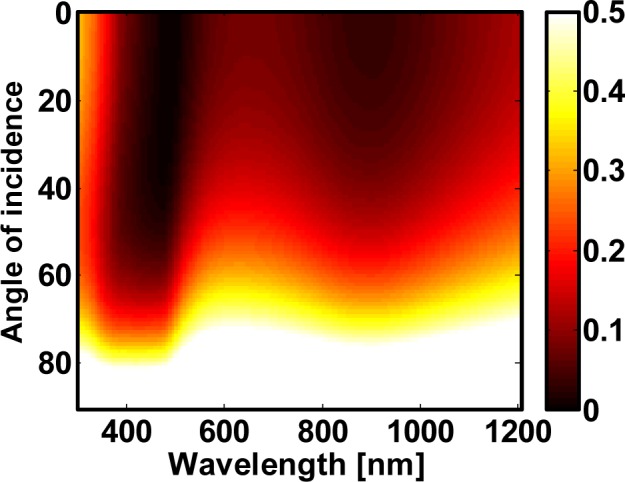
Angle dependent reflection from a silicon substrate that is covered with a dielectric spacer (*ϵ* = 1.69) with a thickness of 70 nm and a layer made from a 20 nm thick gold nanocomposite. Colorbar in the figure is truncated to 0.5 to maintain clarity.

Results of the simulated reflection as a function of the wavelength for selected spacer thicknesses are displayed in Figure 6a. The solid lines are calculated with a thin film transfer matrix routine, where the effective properties of the nanocomposite are taken into account. For each configuration dots correspond to a devoted full wave simulation using the FDTD method. Within these simulations, the plasmonic nanocomposite was considered as it is, *i.e.*, while taking the geometrical details and the presence of the nanospheres fully into account. Perfect agreement in all situations can be seen. This clearly proves that the effective properties of the nanocomposite are reliable. Moreover, the suppression of the reflectance can be also clearly seen when compared to the reflectance of the nanocomposite alone or when compared to the spacer alone as deposited on the silicon substrate that is shown in Figure 6b. Eventually, the anti-reflection action is also largely angle independent as can be seen in Figure 7 in the example of a 70 nm spacer layer. Details on the functionality of the nanocomposites as anti-reflection coatings will be published elsewhere. In general, with this analysis we have shown that plasmonic nanocomposites can be used in the design of optical elements.

## 4. Conclusions

As a short conclusion, we have discussed the effective optical properties of plasmonic nanocomposites with an extremely large filling fraction. The filling fraction is in the region where traditional effective medium theories are no longer applicable. This requires the use of full wave optical simulations. The effective properties show anomalous features, such as the obvious appearance of multiple resonances in the spectrum despite the use of identical nanospheres. The strong horizontal coupling of metallic nanoparticles in the nanocluster has been identified as the reason for these features. We discussed the dependency of effective properties on the filling fraction and on the material from which the nanocomposites are made. Interestingly, nanocomposites made from gold have a stronger dispersion. This is against common wisdom suggesting that gold as a plasmonic material is usually inferior to silver. We have argued here that the stronger intrinsic absorption of gold eventually turns out to be something useful, since it suppresses the interaction between neighboring nanoparticles in the nanocomposite. This suggests that higher filling fractions are feasible without encountering detrimental effects. We wish to stress that similar insights have been obtained while discussing the optical properties of meta-atoms with an electric and magnetic dipole resonance in the transition from a periodic to an amorphous arrangement [43,44]. There, the optical response is invulnerable against disorder in the magnetic resonance, since the resonance is dominated by dissipative losses. In contrast, the optical response changes drastically in the electric resonance, since the resonance is dominated by radiative losses. The stronger the radiative losses are, the more the optical response of amorphous metamaterials degrades, due to the mutual interaction.

Finally, the effective properties of the nanocomposite have been shown to be useful for predicting the optical response of functional optical devices, *i.e.*, here an anti-reflection layer on top of a silicon substrate. This ability opens the door for considering plasmonic nanocomposites comparably to ordinary materials in a future optical design process. This will have implications for many applications, where these nanocomposites may find use in terms of nano-coatings.
